# Simultaneous Detection of Two Chemicals Using a TE_20_-Mode Substrate-Integrated Waveguide Resonator

**DOI:** 10.3390/s18030811

**Published:** 2018-03-07

**Authors:** Ahmed Salim, Muhammad Usman Memon, Sungjoon Lim

**Affiliations:** School of Electrical and Electronics Engineering, College of Engineering, Chung-Ang University, 221, Heukseok-Dong, Dongjak-Gu, Seoul 156-756, Korea; ahmedsalim789@gmail.com (A.S.); musmanm@outlook.com (M.U.M.)

**Keywords:** dual detection, TE_20_-mode substrate-integrated waveguide, microwave sensor

## Abstract

Microwave resonators working as sensors can detect only a single analyte at a time. To address this issue, a TE_20_-mode substrate-integrated waveguide (SIW) resonator is exploited, owing to its two distinct regions of high-intensity electric fields, which can be manipulated by loading two chemicals. Two microfluidic channels with unequal fluid-carrying capacities, engraved in a polydimethylsiloxane (PDMS) sheet, can perturb the symmetric electric fields even if loaded with the two extreme cases of dielectric [ethanol (E), deionized water (DI)] and [deionized water, ethanol]. The four layers of the sandwiched structure considered in this study consisted of a top conductive pattern and a bottom ground, both realized on a Rogers RT/Duroid 5880. PDMS-based channels attached with an adhesive serve as the middle layers. The TE_20_-mode SIW with empty channels resonates at 8.26 GHz and exhibits a −25 dB return loss with an unloaded quality factor of Q ≈ 28. We simultaneously load E and DI and demonstrate the detection of the four possible combinations: [E, DI], [DI, E], [E, E], and [DI, DI]. The performance of our proposed method showed increases in sensitivity (MHz/ε_r_) of 7.5%, 216%, and 1170% compared with three previously existing multichannel microwave chemical sensors.

## 1. Introduction

The use of multiple sensors is important for several applications, including the monitoring of chemical parameters in complex specimens and samples (such as blood), aerodynamic research, the pharmaceutical and chemical industries, and quality control of food [1]. Using multiple sensors or a sensor array is not an obvious choice because of their large footprint, high power consumption, and the complexity involved in their design and fabrication for mass production. A single-chip sensor that achieves simultaneous multiple detections can be visualized as an alternative approach and is the prime objective of this study. 

The simultaneous detection of multiple chemicals has been a fascinating research area for engineers, especially for personnel involved in analytical chemistry. Microfluidics can be used to screen multiple liquids simultaneously, an idea anticipated by Whitesides in 2006 [2]. In 2010, optical chemical sensors based on stimuli-responsive materials were combined for monitoring multiple analytes such as (1) carbon dioxide and oxygen and (2) pH and temperature [1]. In the same year, in another study, a single sensor for monitoring multiple analytes by exploiting two detection modes was developed [3]. The selective binding of cations or anions was monitored by the changes in the photophysical properties of receptor sites via either UV-vis (for iodide ions) or fluorescence spectroscopy (for Fe^3+^). In 2012, an electronic nose built from a metal-oxide chemiresistor array was developed to sense multiple biomarkers in breath [4]. Pure biochemical sensors (non-electromagnetic) exhibit excellent sensitivity and targeted selectivity. However, biologically analyzing systems and sensors requires sophisticated equipment for processing (e.g., fluorescence labeling), laborious sample preparation, and/or offsite processing for results verification [5]. Therefore, they are complex and expensive.

Radio Frequency (RF) resonators using patch antennas, ring resonators, substrate-integrated waveguides (SIWs), and metamaterial elements have been proposed as biochemical sensors when integrated with microfluidic channels [6]. The sensitivity of a commercial product (sensor) is provided in terms of its limit of detection (LOD), which is the lowest concentration of the target analyte that a chemical sensor can reliably detect with repeatability. RF/microwave chemical sensors have shown sensitivities of 7890 ppm [7] and 78,990 ppm [8], whereas non-electromagnetic sensors have exhibited sensitivities in the order of tens of ppm [9,10] or even lower. Although RF sensors are roughly 1000 times less sensitive than their non-electromagnetic counterparts, their merits, such as their miniaturized size, light weight, being contactless, and low cost, make them attractive for further research and investigation. They require microliter–nanoliter sample volumes, which is an advantage with precious fluids, for instance, blood. The noncontact feature of these sensors is considered important for the screening of biochemical fluids, as it prevents contamination of the target analyte. Despite showing adequate sensing capabilities, pure RF chemical sensors are unable to exhibit selectivity toward a specific analyte, and this is still a challenging research area. Moreover, to date, we have not witnessed the simultaneous sensing of multiple liquids using a single RF chip (in a non-array configuration).

Metamaterial (MM) elements are artificially synthesized periodic structures [11] that show negative permittivity and permeability simultaneously [12,13]. Aside from the smaller footprints of MMs compared with SIWs, they provide a high quality factor (Q) and, therefore, have been an attractive choice for realizing sensing devices [14,15]. Their geometry, shape, orientation, and properly applied electric/magnetic field determine their usefulness and operating frequency [16,17]. Split ring resonators (SRRs) and complementary split ring resonators (CSRRs) are the most utilized topologies of metamaterials for developing a variety of RF circuits and components [18,19,20,21]. Next, we highlight existing RF resonators proposed for the simultaneous detection of multiple fluids. 

A multichannel resonator was proposed as a chemical sensor in [22]. Four SRRs of different dimensions, coupled with a microstrip line, were developed to observe four unique resonance frequencies, which were independently tuned by loading 5 µL of ethanol on the split gap of each SRR. In [23], a microfluidic multichannel array based on three open SRRs of different dimensions was proposed. To demonstrate the possibility of dual detection, one of the fluids was held constant, while the other was varied. This variations in permittivity validated the independent tuning of the corresponding resonance frequencies. In [24], an ingenious design was proposed for noncontact dual detection of chemicals. Two SRRs coupled with a microstrip line were realized using the frequency splitting phenomenon (by loading two asymmetric dielectric loads), which led to two unique resonance frequencies. Two polydimethylsiloxane-based (PDMS-based) microfluidic channels were loaded on the split gap of the SRRs. Deionized (DI) water was maintained in one channel as a reference liquid, and ethanol loading in the other channel caused a frequency shift of 70 MHz. When DI water was used in both channels, no frequency shift was observed, as expected.

SIWs are a modern form of waveguide technology that has outperformed others in various RF applications [25,26]. The propagation characteristics of an SIW structure are similar to those of a conventional waveguide as long as the metallic vias are closely spaced and radiation leakage can be neglected [27]. The transverse electric (TE) modes of rectangular metallic waveguides also exist in SIWs. Transverse magnetic (TM) modes are not supported by SIWs because of the gaps between the metal vias [27]. The high electric field energy concentrated in the SIW cavity has been exploited in a variety of sensing applications [7,25,28,29]. Its microstrip-like planar structure and its ability to preserve the advantages of rectangular waveguide technology (low loss and high Q) make it a favorite choice for sensing applications. It has additional significant advantages, such as a low cost, compact design, and simple fabrication process. Loss minimization in SIWs is an important factor, which can be controlled by the choice of substrate (dielectric properties) and by geometry-dependent parameters of the SIW structure. These are discussed in the design section.

State-of-the-art chemical sensors based on the SIW structure are discussed in this paragraph. In [30], a high quality factor (Q ≈ 300) SIW was proposed as a microwave humidity sensor. The functionalized region was introduced on the highest E-field region using air holes. A larger functionalized area ensured more interaction with the moisture in the air via and, hence, the sensitivity was increased. Another remarkable feature was enhanced sensitivity without using any coating material, unlike existing humidity sensors. In [31], an SIW resonator (with TE_101_ and TE_102_ modes) functionalized with tin oxide (SnO_2_) powder was proposed to detect hydrogen gas. The influence of the size and topology of the functionalized area, substrate permittivity, and grain size of the micro powder (tin oxide) on the sensitivity of the device was investigated. Size reduction of an SIW without compromising performance was attempted using different techniques and structures such as folded SIWs, ridged half-mode SIWs (RHMSIWs), quarter mode SIWs (QMSIWs), ridged quarter-mode SIWs (RQMSIWs), and eight-mode SIWs (EMSIWs) [32]. Conforming to image theory, the in-phase electric fields exist on opposite sides around the magnetic wall of an SIW [26]. The half-mode SIW (HMSIW) and QMSIW can maintain the same field distribution as regular SIWs, while bringing down the size of the design to one half and one fourth, respectively [19,33]. In [32], an RQMSIW resonator was proposed as a humidity sensor. The unloaded quality factors were measured to be 35 and 86 for the QMSIW and RQMSIW, respectively. Its area reduction, compared with the QMSIW, was approximately 48%, while the measured sensitivity (30.73 kHz/RH) was almost the same as that of the QMSIW (36.50 kHz/RH). An EMSIW antenna has been proposed as a miniaturized chemical sensor, and has shown a frequency shift of 70 MHz with respect to DI water and ethanol [7]. In [28], a rectangular SIW cavity was proposed in order to characterize several liquid chemicals, and a frequency shift of 610 MHz was observed. In [34], a circular SIW cavity (Q ≈ 1080) was proposed as an ethanol sensor, and it showed a frequency shift of 380 MHz. There was a significant improvement in the sensing performance or otherwise miniaturization in each of these SIWs; however, all these sensors had the same limitation of only single-channel sensing per sensor.

The internet of things (IoT), which is a rapidly growing technology, is associated with enormous sensing capabilities attached to many gadgets, devices, and consumer electronic products. The frequency ranges used in IoT are likely to be in the high MHz to the low GHz range, which allow for the operation of short range communication protocols such as Bluetooth, Zigbee, Wi-Fi, and Near Field Communication (NFC). However, because of the existing frequency interference on crowded RF channels (physical layer), the use of high frequency spectra (such as the C band in the 4–8 GHz range and the X band in the 8–12 GHz range, as per the IEEE definition) can be a new paradigm to be included in IoT. Moving the carrier frequency toward the C and X bands can bring advantages, such as miniaturized devices and systems. Our proposed dual-detection RF chemical sensor, which operates in the range of 7–8 GHz, can be a potential candidate in such a scenario.

This paper proposes a TE_20_-mode SIW resonator that can simultaneously detect two analytes using a single-chip sensor. Exploiting two distinct yet symmetric electric field regions, two microfluidic channels with an unequal fluid-carrying capacity are engraved in PDMS to be embedded in a sandwich-like structure. We achieved four unique resonance frequencies corresponding to four combinations of ethanol and DI water, alternately loaded in the channels. Alternatively, the sensing of a single analyte using multiple channels allows for more reliable detection, compared with the sensing of a single analyte using only one channel. In this paper, we explain the design guidelines and the simulation process of the proposed design carried out to obtain our results. After making some comparisons with previous multichannel RF sensors, merits and demerits that are worth mentioning are discussed.

## 2. Sensor Design

### 2.1. Theory

Rectangular waveguide resonators confine electromagnetic fields inside a localized area, and thus eliminate radiation losses and high-resistance effects [35]. Cavity resonators (not related to the SIW cavity) support wave propagation in both TE and TM modes, just like rectangular waveguides [35], and the TE_10_ and TE_20_ modes are, respectively, the dominant and second-order modes in these resonators. Rogers Corporation Inc. (Chandler, AZ, USA) has been manufacturing low-loss dielectric substrates specially designed for passive circuits and components operating in the microwave and millimeter-wave frequency ranges. They are widely utilized because of their highly stable dielectric properties [24,36]. RT/duroid 5870 and 5880 laminates provide low dissipation factors, such as 0.0012 and 0.0009, which makes them particularly useful in the Ku-band (12–18 GHz) and above. These laminates are resistant to all solvents and reagents normally used when etching printed circuits or plating edges and holes [37]. If the height (thickness of a Rogers/RT Duroid 5880 substrate: *h_sub_* = 0.51 mm) of an SIW cavity resonator is much smaller than its length (*L_SIW_*) and width (*W_SIW_*), the resonant frequency (*f_r_*) of an SIW cavity can be defined using the following expression [28]:

(1) $${\left(fr\right)}_{mn}=\frac{1}{2\pi \sqrt{\mu \varepsilon }}\sqrt{{\left(\frac{m\pi }{W_{SIW}}\right)}^{2}+{\left(\frac{n\pi }{L_{SIW}}\right)}^{2}},[\mathrm{Hz}]$$

where *m* and *n* represent the integer-mode indices and *µ* and *ε*, respectively, represent the permittivity and permeability of the dielectric material. The resonant frequency depends upon the length, width, and dielectric constant of the substrate. For TE_20_-mode propagation, the equation can be reduced to the following form [35]:

(2) $${\left(fr\right)}_{20}=\frac{c}{W_{SIW}}$$

where *c* has a value of 3 × 10^8^ m/s.

**Figure 1:**
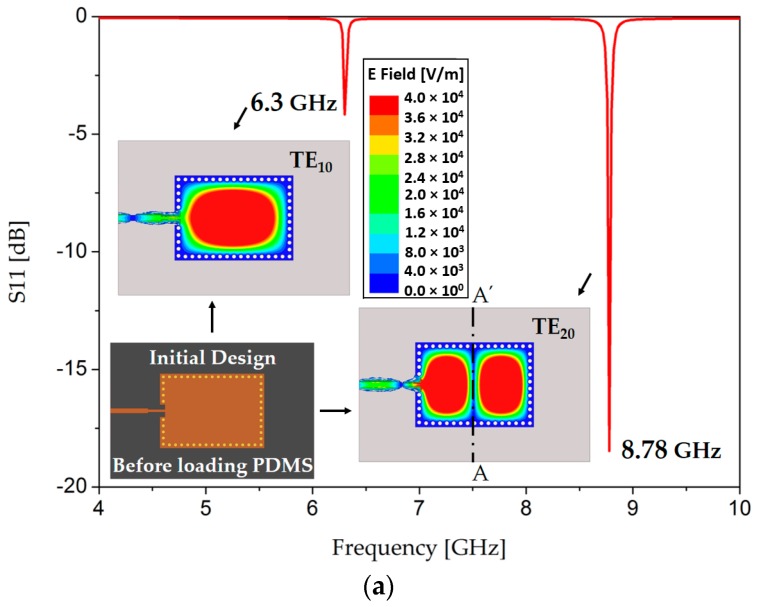

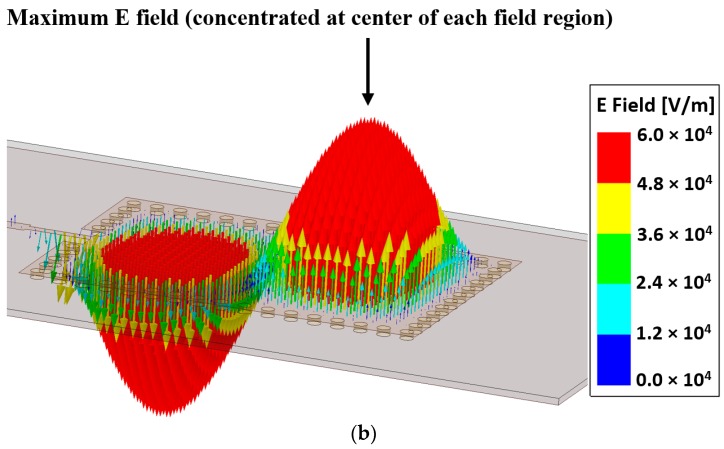
(**a**) Electric field magnitude of the TE_20_-mode SIW resonator, showing the potential for dual-detection sensing; (**b**) Electric field vector distribution of the TE_20_-mode SIW resonator, creating expectations for a strong interaction of electromagnetic (EM) waves with the microfluidic channels. The vertical orientation of the EM waves suggests that the microfluidic channels should be placed horizontally.

Before delving into design details, we present the electric field magnitude of an initially drawn TE_20_-mode SIW, because it is the main motivation that gave rise to our concept of dual detection. The structure shows resonance frequencies at 6.3 and 8.78 GHz for the TE_10_ and TE_20_ modes, respectively, as shown in Figure 1. This figure (E field in TE_20_ mode) shows that two distinct electric field regions are symmetric around the center line AA’, which can be used to construct a dual-detection sensor using the TE_20_-mode SIW resonator. The electric field vector distribution is odd symmetric with time reversal [38,39], as shown in Figure 1b.

**Figure 2:**
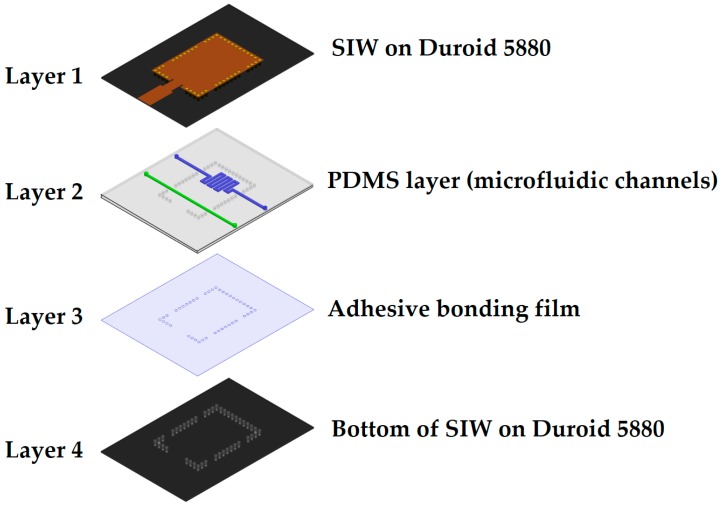
Stacked-layer structure of the TE_20_-mode SIW resonator with the microfluidic channels as the middle layer.

The stacked-layer structure of our proposed dual-detection chemical sensor is shown in Figure 2. The top and bottom layers are represented by a conductive pattern and the ground of the SIW structure, respectively. A PDMS layer attached to an adhesive bonding film serves as the middle layer in this sandwich-like structure.

### 2.2. Design of the Dual-Detection Chemical Sensor

Now, we explain the simulated design process of our proposed resonator. The conductive SIW rectangular patch and ground (a × b = 60 mm × 40 mm) are realized on the top and bottom layers of a low-loss Rogers RT/Duroid 5880 substrate (ε_r_ = 2.2 ± 0.02, tan Δ = 0.0009, and h_sub_ = 0.51 mm). The size of the SIW patch (c × e = 30.8 mm × 22.2 mm) was chosen so that it resonates around 8 GHz and is fed by a combination of an inset-feed quarter-wave transformer and a microstrip line. Shorting vias (silver material) are inserted along the boundary of the patch to connect the top (patch) and bottom ground to realize a magnetic sidewall along the patch. This virtual wall preserves the electric field inside the patch, which can be used for sensing applications. The width of the microstrip line was designed in accordance with a 50-Ω SubMiniature version A (SMA) connector. The diameter of the vias (*d*) and the center-to-center spacing between consecutive vias (pitch *p*) are relevant parameters to minimize losses and improve the SIW performance, and are given as $d<\frac{\lambda_{g}}{5}$ and p<2d [27], where λ_g_ is the guided wavelength. 

**Figure 3:**
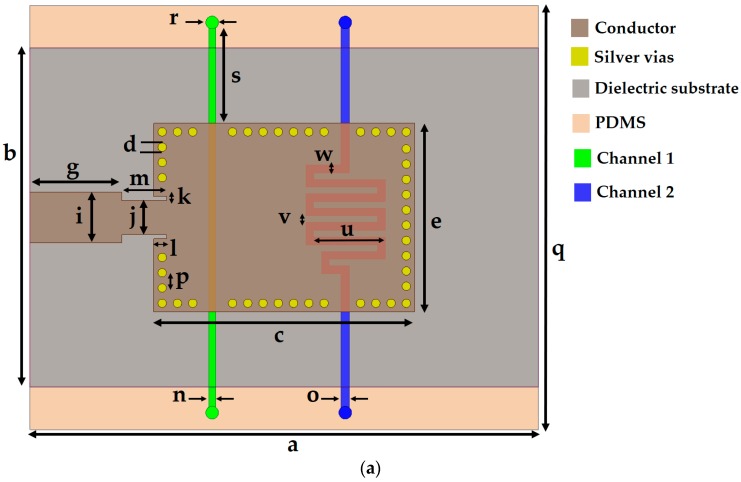

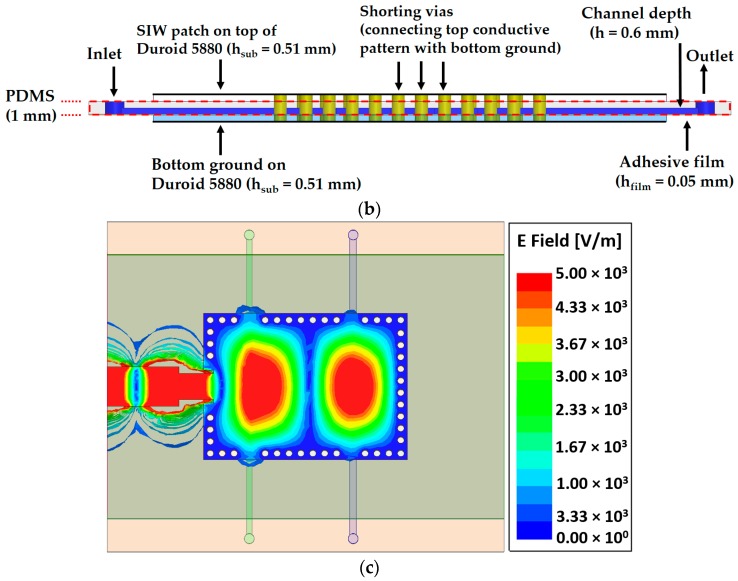
(**a**) Layout of the TE_20_-mode SIW resonator proposed as a dual-detection chemical sensor; (**b**) Side view of our proposed sensor. Liquid flows in the PDMS-based channels, while the bottom of the PDMS layer is covered using an adhesive bonding film, giving it its “noncontact” features. The fluid channel depth inside the PDMS layer is represented in a blue color; (**c**) Electric field distribution of the TE_20_-mode SIW resonator resonating at 8.28 GHz (with both channels empty).

To perturb the effective permittivity of the dielectric substrate, the microfluidic channels are placed at such a position so that they overlap at the region with the highest electric field. Two microfluidic channels of depth h = 0.6 mm were designed in a 1-mm-thick PDMS layer (ε_r_ = 2.7 and tan Δ = 0.05) [8]. To avoid direct contact of the chemical with the substrate, a two-sided adhesive film (ε_r_ = 3.4, tan Δ = 0.03, and h_film_ = 0.05 mm) was placed below the PDMS layer (as shown in Figure 2), and these two middle layers were inserted between the top and bottom Rogers substrates. Once the PDMS layer was inserted and the microfluidic channels were loaded with chemicals, the impedance matching was disrupted and some design parameters (e.g., width of the microstrip line, dimensions of the quarter-wave transformer, inset feed, and via adjustment near the microfluidic channel) needed to be changed. The width of the microstrip line (i = 6 mm) was readjusted for 50-Ω matching because the thickness of the structure changed after the insertion of the PDMS layer between the two Rogers substrates. A quarter-wave transformer with inset feed (l × k = 1.5 mm × 0.5 mm) was used to match the impedance of the SIW patch to a 50-Ω microstrip line. The dimensions of the quarter-wave transformer (m × j = 5.25 mm × 4 mm) were determined via parametric simulation to achieve a good impedance match using an ANSYS high-frequency structural simulator. The final layout of our proposed TE_20_-mode SIW resonator with embedded microfluidic channels is shown in Figure 3, and all design parameters are listed in Table 1.

### 2.3. Design and Motivation for Asymmetric Microfluidic Channels

**Figure 4:**
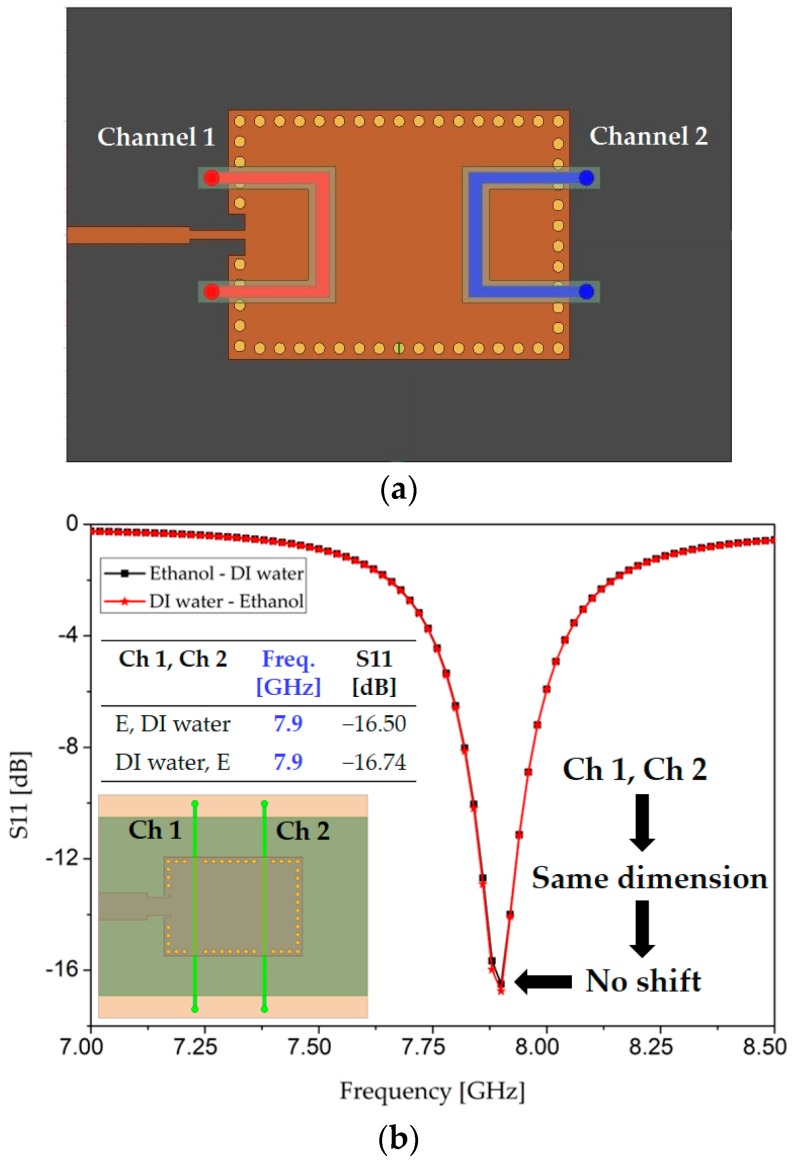
(**a**) Top view of the TE_20_-mode SIW resonator with two PDMS-based microfluidic channels as the top layer. This configuration is unable to show distinct resonance frequencies for the two extreme cases of dielectric loading, namely [ethanol, DI water] and [DI water, ethanol]; (**b**) Simulated S11 when two symmetric microfluidic channels (placed as the middle layer) are loaded with the extreme cases in our TE_20_-mode SIW resonator. “E” is the naming convention of ethanol in this manuscript.

Two microfluidic channels are required to simultaneously load two chemicals for assessing the proposed dual-detection sensor. First, a PDMS layer (containing two microfluidic channels) was loaded on top of the two distinct electric field regions to investigate if there was a reasonable frequency shift. However, the extreme cases of [ethanol, DI water] and [DI water, ethanol] exhibited the same resonance frequency, so the idea of using PDMS as the top layer was rejected (see Figure 4a). The next option was to embed PDMS as a middle layer.

First, we shall explain the motivation behind using two asymmetric microfluidic channels. We initiated our design with two simple straight microfluidic channels having the same dimensions (i.e., length, width, and thickness, *h*), designated as Ch 1 and Ch 2, as shown in the inset of Figure 4b. We selected ethanol and DI water as the target analytes owing to their widely known dielectric properties. When the dielectric properties of the two extreme cases of [ethanol, DI water] and [DI water, ethanol] were simulated on these symmetric channels, the resonance frequency remained exactly the same in both cases, as can be seen in Figure 4b, despite the widely different dielectric constants of ethanol (ε_r_ = 5, tan Δ = 0.41) and DI water (ε_r_ = 67, tan Δ = 0.38) [40]. The absence of frequency shifts can be explained by the fact that a change in the effective dielectric constant was zero in the cases of [ethanol, DI water] and [DI water, ethanol]. They uniformly perturb the symmetric E-field regions, which results in zero shift. To mitigate this issue, we envisaged using two asymmetric channels, i.e., channels with unequal fluid-carrying capacities. To keep the channel design simple yet sensitive, we thought that one channel (e.g., Ch 1) could be a simple straight stream, while the other (Ch 2) could have any defined shape, such as a circular or meander shape. Unlike Ch 1, a large overlap with a high-intensity electric field region in the case of Ch 2 was expected to non-uniformly disrupt the corresponding electric field region. From our previous experience (measurements), air bubbles hinder the uniform filling of a circular channel with liquid, especially when a micropipette or syringe is used for liquid injection instead of a micropump [28]. Therefore, we designed one channel as straight and the other one with a meander shape and tested them by loading them with the two similar cases of [ethanol, DI water] and [DI water, ethanol]. Meander patterns increase the equivalent inductance and consequently the resonance frequency is decreased [41,42]. After considering the fabrication limits and sensitivity, a meander-shaped channel with six bends was designed. There was a significant shift in the resonance frequency (we will demonstrate each case in the next section). From the channel dimensions shown in Figure 3, we can estimate their fluid-carrying capacities, which are 21.36 µL for Ch 1 and 48 µL for Ch 2. 

### 2.4. Optimized Geometry and Shape of the Microfluidic Channels

**Figure 5:**
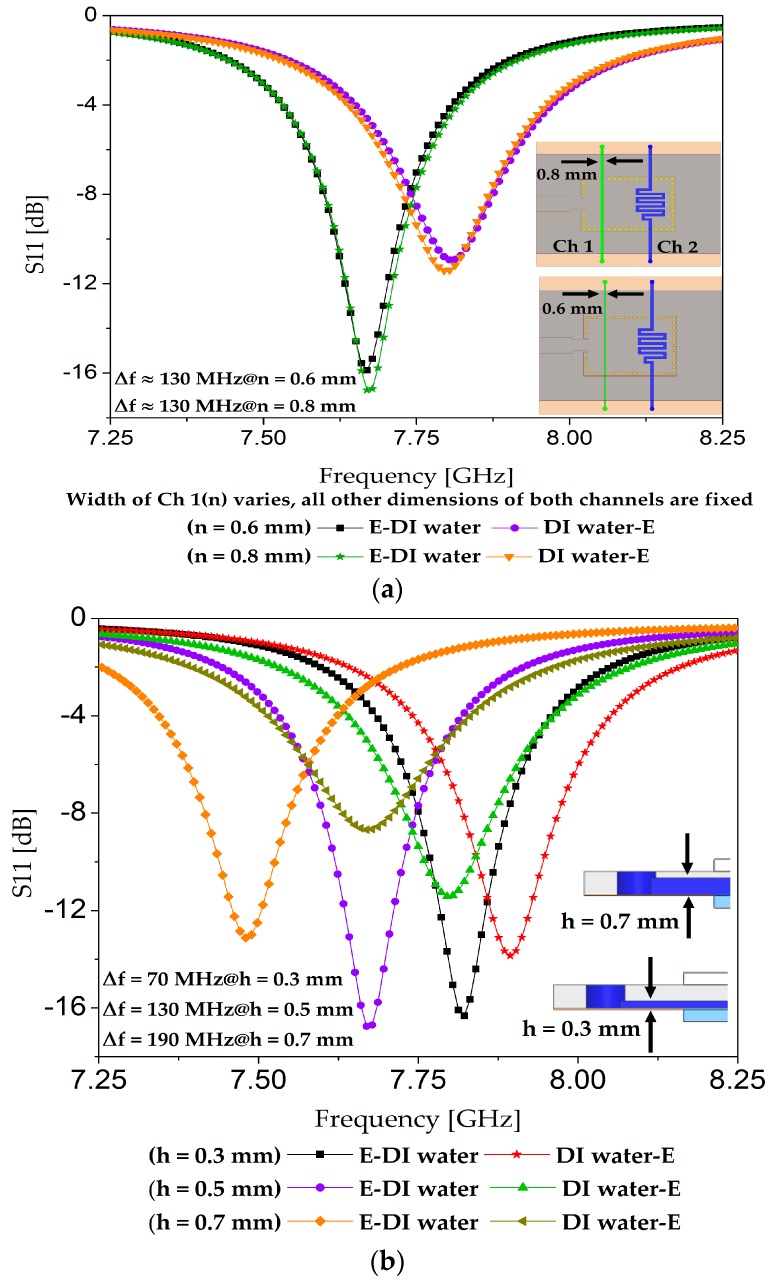
(**a**) Variations in the width of Ch 1 (represented by *n*) to try to obtain the maximum value of frequency shift (Δ*f*). The two extreme cases of [ethanol, DI water] and [DI water, ethanol] were loaded for each value of *n*, and the resultant Δ*f* is illustrated. All the other parameters, such as length and depth (h = 0.5 mm) of both channels, were fixed; (**b**) Variations in channel depth (h) to try to obtain the maximum value of frequency shift (Δ*f*). The two extreme cases of [ethanol, DI water] and [DI water, ethanol] were loaded for each value of *h*, and the resultant Δ*f* is illustrated. All the other parameters, such as length and width of both channels, were fixed, as mentioned in Figure 3a and shown in the inset of this figure.

To optimize our design, different widths and thicknesses of the two channels (straight and meander-shaped) were investigated. The optimization criterion during all these simulations was achieving the maximum frequency shift possible when loading the extreme cases of [ethanol, DI water] and [DI water, ethanol]. For instance, *n* = 0.8 and 0.6 mm were tested as the width of the straight channel, as shown in Figure 5a. Both values yielded almost the same performance (frequency shift), and it can be surmised that a small step variation (0.2 mm) in the width of Channel 1 is unable to cause a significant difference in the resonance frequency because of the dominant effect of Channel 2, owing to its higher overlapped area with the electric field region. We selected *n* = 0.8 mm, taking ease of fabrication into consideration. In another set of simulations, the thickness of both channels was set as 0.3, 0.5, and 0.7 mm, to try to obtain the maximum frequency shift by loading the extreme cases. We observed an increase in the frequency shift when loading [ethanol, DI water] and [DI water, ethanol] when the channel thickness (*h*) was increased, as seen in Figure 5b. Note that Figure 3a,b show the top view and side view of our final design. 

## 3. Simulation Analysis

**Figure 6:**
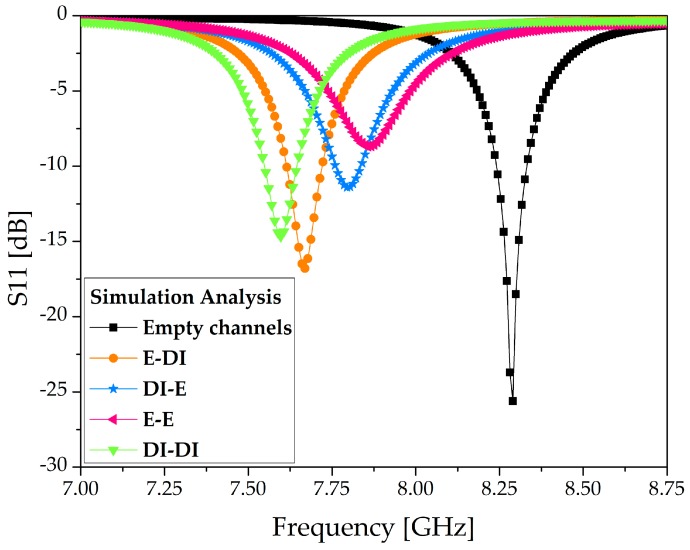
Simulation S11 of the TE_20_-mode SIW resonator proposed as a dual-detection sensor. E and DI are the naming conventions for ethanol and deionized water, respectively.

We simulated the microfluidic channels using four configurations of ethanol and DI water, and the S-parameters of our proposed sensor are presented in Figure 6. The dielectric properties of PDMS were set as ε_r_ = 2.7 and tan Δ = 0.05 [8]. When the channels are empty, the structure resonates at 8.26 GHz. Using full-wave simulation, four distinct resonance frequencies, 7.66, 7.79, 7.85, and 7.59 GHz, were achieved when [ethanol, DI water], [DI water, ethanol], [ethanol, ethanol], and [DI water, DI water] were loaded on the microfluidic channels. The resonance obtained with [ethanol, ethanol] showed the lowest Q factor, which is attributable to it being the loading configuration with the highest losses among the four configurations used. For this full-wave simulation, the dielectric properties of 100% ethanol were set as ε_r_ = 5.2 and tan Δ = 0.4 [43]. The simulated unloaded quality factor Q ≈ 28 was calculated based on the well-known formula as specified in [34,44].

## 4. Fabrication and Measurement

### 4.1. Fabrication

**Figure 7:**
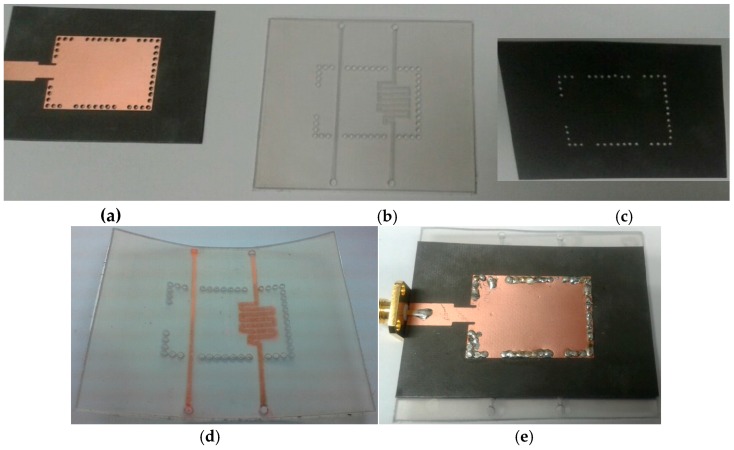
Fabrication phases of the TE_20_-mode SIW resonator proposed as a dual-detection sensor: (**a**) Top SIW layer realized on Duroid 5880; (**b**) PDMS layer containing the two microfluidic channels; (**c**) Bottom layer of the SIW realized on Duroid 5880; (**d**) Colored water (red) injected into the microfluidic channels to verify a uniform and smooth filling; (**e**) Final prototype after inserting vias and soldering.

The proposed sensor was realized using four layers, as shown in Figure 7. The top and bottom layers, respectively, were comprised of an SIW conductive pattern and ground, and were fabricated by conventional photolithography on the aforementioned Rogers RT/Duroid 5880 substrate. The microfluidic channels were engraved in a PDMS sheet using a laser cutting machine. Channel etching using a laser cutting machine is a quick and straightforward process compared with soft lithography and mold casting techniques. However, the latter produces nano-level surface finishing at the cost of complexity. If fine surface finishing and complex 3D features are not required, then a laser cutting machine serves satisfactorily for small-scale designs, for instance, laboratory level designs or prototype manufacturing. A two-sided adhesive film, ARcare^®^ 92848 (h_film_ = 0.05 mm, provided by Adhesives Research, Inc., Glen Rock, PA, USA [45]), was attached below the PDMS layer. In addition to bonding between PDMS and the bottom Duroid, it prevented the bottom substrate from coming into direct contact with the liquids. The simultaneous drilling of holes in all layers for via insertion eliminated the possibility of misalignment issues. NanoPort™ fittings (Coned Port Version) provided by IDEX Corporation (Lake Forest, IL, USA) facilitated the injection and removal of the analytes [46]. To place the NanoPort fittings inside the PDMS layer, holes with a depth of 1 mm and a diameter of 1.5 mm were drilled after considering the tip of the coned nut. Pristine ethanol (part number: 32205) was purchased from SIGMA-ALDRICH Korea Ltd. (Yongin-si, Gyeonggi-do, Korea). The stepwise fabrication of the prototype sample is shown in Figure 7.

### 4.2. Measurement

**Figure 8:**
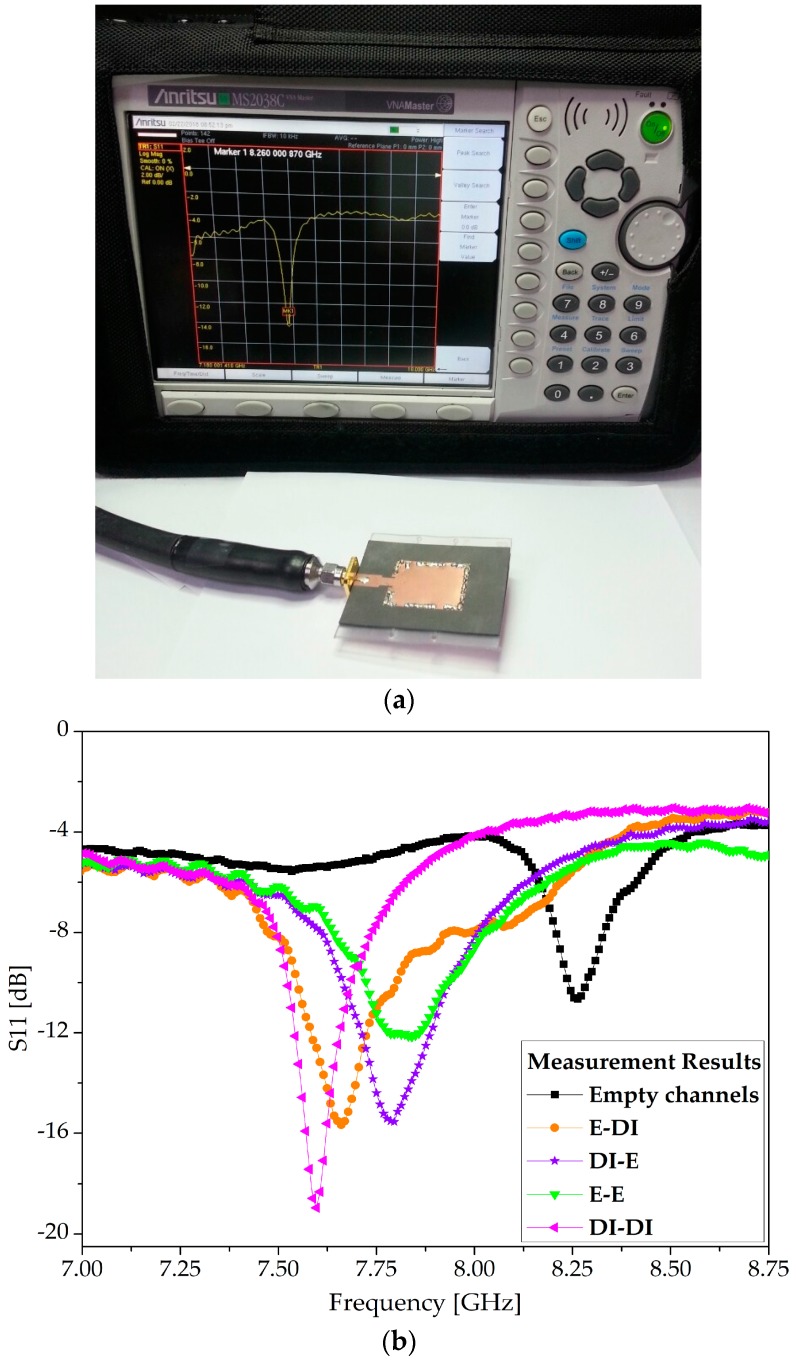
(**a**) Measurement setup: the proposed TE_20_-mode SIW resonator (with both channels unloaded) is attached to a vector network analyzer (Anritsu MS2038C, manufactured by Anritsu Corporation Kanagawa Prefecture, Japan) and resonates at 8.26 GHz; (**b**) Measurements results (S11) when both channels were empty and the four possible combinations of ethanol and DI water were alternately loaded.

**Figure 9:**
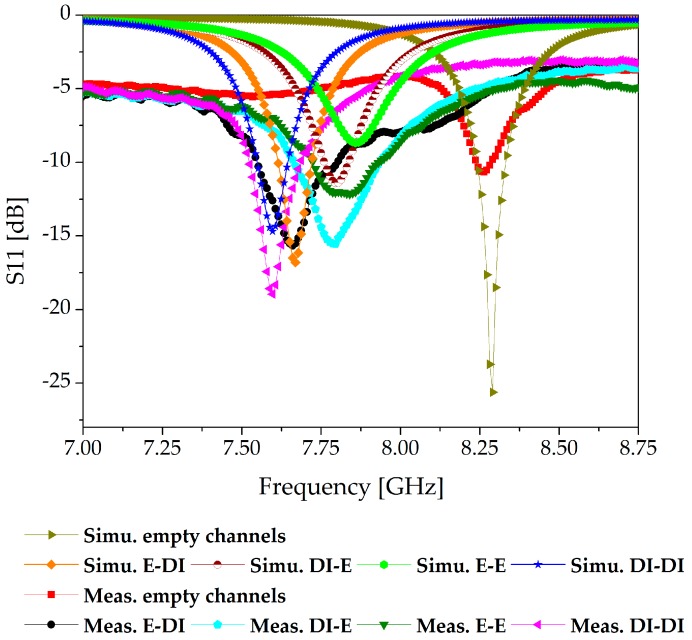
Comparison of the simulation and measurement results when the channels were empty and when the four possible combinations of ethanol and DI water were alternately loaded.

First, S11 measurements were conducted with empty channels, and then the four possible combinations of ethanol and DI water were injected into the channels. After each measurement, the channels were cleaned by exerting air through a syringe and reinstating the reference frequency originating from the empty channels. The effective permittivity of the dielectric changed each time Ch 1 and Ch 2 were filled with any of the [ethanol, DI water], [DI water, ethanol], [ethanol, ethanol], and [DI water, DI water] configurations. The measurement setup and S-parameter measurements are provided in Figure 8. The simulation and measurement results were found to be in good agreement, as shown by the comparison presented in Figure 9. The resonance frequency with both channels empty was measured to be 8.26 GHz. The resonance frequency switched to 7.66, 7.795, 7.84, and 7.60 GHz when the four configurations of [ethanol, DI water], [DI water, ethanol], [ethanol, ethanol], and [DI water, DI water] were injected into the channels, respectively.

The simulation and measurement results of both the resonance frequencies and return losses are summarized in Table 2. To evaluate the precision of these measurements, the relative error in the resonance frequency (*f_r_*) was calculated, which can be defined as:

(3) $$\mathrm{Relative}\;\mathrm{Error}=\left|\frac{\mathrm{actual}\;\mathrm{value}-\mathrm{measured}\;\mathrm{value}}{\mathrm{actual}\;\mathrm{value}}\right|\times 100,$$

where “actual values” represent the simulation values and “measured values” are the values obtained from the fabricated prototype sample of the proposed SIW resonator.

### 4.3. Sensitivity Evaluation

**Figure 10:**
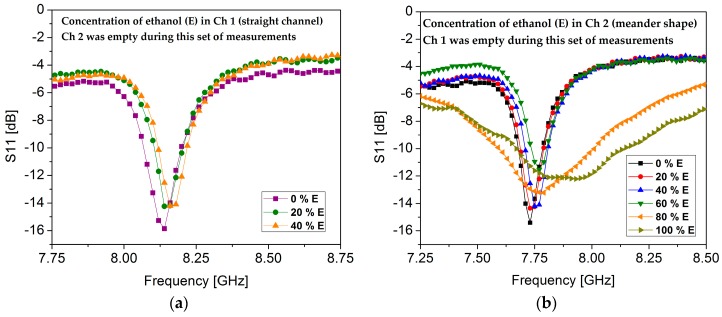
The measured concentrations of ethanol (E) in each channel were analyzed to determine the sensitivity in terms of limit of detection (LOD). (**a**) Concentrations of ethanol in Ch 1 (straight channel) while keeping Ch 2 empty. The LOD of Ch 1 was measured to be 30% ethanol; (**b**) Concentrations of ethanol in Ch 2 (meander shaped) while keeping Ch 1 empty. The LOD of Ch 2 was measured to be 10% ethanol.

Figure 10 shows the measured concentrations of ethanol in each channel while keeping the other channel in an empty state. To determine the limit of detection (LOD), the sensitivity of each channel was independently evaluated. Ch 1 (straight channel) exhibited the same resonance frequency (8.14 GHz) when 0 % ethanol and 20 % ethanol was injected in it while keeping Ch 2 (meander shape) empty. Then, 40 % ethanol was injected in Ch 1, which showed a resonance frequency of 8.16 GHz; the results are summarized in Figure 10a. Ch 1 showed an LOD of 30 %, which is a very low value. However, this was expected because of Ch 1’s small overlapping area with the high E-field region. To determine the sensitivity of Ch 2, various concentrations of ethanol were injected in Ch 2 while keeping Ch 1 empty. Ch 2 showed an LOD of 10%; the measured results are shown in Figure 10b.

To validate our proof of concept, the performance of the proposed dual-detection chemical sensor was compared with other SIW bio/chemical sensors (see Table 3). We analyzed the maximum frequency shift (Δf_max_) of each device irrespective of the amount of change in permittivity. The proposed sensor shows the highest value of Δf_max_ and provides a platform for the simultaneous detection of two chemicals. As shown in Table 3, single-channel sensing is a major limitation in other SIW chemical sensors. Further advantages of multichannel sensing are explained in the Discussion section.

To make a fair comparison, the performance of our proposed dual-detection chemical sensor was compared with that of already proposed dual/multiple-detection RF chemical sensors (see Table 4). To evaluate the performance of all sensors, the fractional variations in resonance frequency with respect to effective permittivity were calculated, which can be defined as S = (Δf/ε_r_), where ε_r_ = ε_a_ − ε_air_, with ε_a_ and ε_air_ representing the permittivity of the analyte and air, respectively. The unit of sensitivity is MHz/ε_r_. We also compiled a qualitative analysis of our proposed sensor with existing dual/multiple-detection sensors (see Table 5). The performance of our proposed sensor showed increases in sensitivity (MHz/ε_r_) of 7.5%, 216%, and 1170% compared with three existing multichannel RF chemical sensors. Comments about the results presented in Table 4 and Table 5 are presented in the Discussion section.

## 5. Discussion

In this section, the merits and demerits of our proposed sensor are critically discussed, and the contributions made to solve the current issues of multichannel RF chemical sensors are explained.

As a widespread practice in RF microfluidic sensors, PDMS channels are manually aligned onto the highest sensitivity area, which is usually a narrow region. However, misalignments when positioning the channel are liable to influence measurement accuracy and sensor performance [49]. In general, microfluidic channels are adhesively attached to a dielectric substrate or conductive pattern as a separate layer, potentially giving rise to another instance of misalignment [49]. Our proposed TE_20_-mode SIW resonator used as an ethanol sensor eliminates the need to align the PDMS channel with the Rogers substrate because the stacked layers have concurrently aligned vias.

In conventional RF resonators using a single channel for sensing, such as those presented in [7,28,34,47] (a comparison has been already presented in Table 3), if the channel is biased due to any reason (such as misalignment or fabrication error), which is a common situation when handling high-frequency circuits or stacked-layer devices in which microfluidics are attached as a separate layer, the sensing measurements may become unreliable. Considering a similar situation (channel biasing) in a dual-channel sensor, using one channel (liquid) as a reference and the other channel for sensing an analyte can provide more reliable detection, compared with using a single channel per sensor [50,51].

First, we will discuss the present issues in existing multichannel RF chemical sensors, and then we will explain how our proposed sensor partially addresses these concerns. Without a doubt, in [22], a multichannel chemical sensor with independent tunability was achieved; however, in that study, microfluidic channels were not used, whose absence can cause twofold liabilities—lack of reusability and risk of erroneous measurements—if the sample is contaminated with pollutants from the air or the environment or if the target analyte is not loaded at the highest sensitivity area (which is a very narrow region, in the order of 0.15 mm). These issues were solved by the authors of [23], who proposed a microfluidic multichannel array. However, only one of the three resonance frequencies was independently tunable by keeping one liquid constant. In addition, the simultaneous loading and detection of more than two liquids was not possible. Moreover, they neither discussed the independent tuning of the three resonance frequencies nor mentioned any relevant measurements. In [24], a noncontact dual-detection chemical sensor was proposed. The reference liquid (DI water) was fixed in one channel, and the other channel could be loaded with liquids with a permittivity different from the reference liquid. In the case of DI water being used in both channels, no frequency shift was observed, as expected. Therefore, the dual-detection capability of this design cannot be considered independently tunable.

Our proposed sensor exhibited a single resonance frequency controlled by the dielectric loadings of the two channels; therefore, it also cannot be considered to be independently tunable. However, unlike the sensor presented in [22], microfluidic channels were utilized in our proposed sensor for dual detection. The utilization of microfluidic channels resolves the issues that arise from the absence of microfluidics. On the other hand, unlike [24], a unique resonance frequency is obtained in our proposed sensor for dual sensing if the two channels are loaded with liquids of the same permittivity.

RF/Microwave resonators used as chemical sensors are unable to exhibit selectivity toward any target analyte, which constitutes a fundamental drawback that is still unresolved. Unresponsiveness toward interfering particles in a sample matrix is called “selectivity”, and a lack of it in RF chemical sensors is a serious issue if they are to be utilized as a marketable product [36]. Hybrid sensing, using RF sensors with an additional layer of chemical coating, provides selectivity up to some extent. For instance, refer to [51,52,53]. In [51], a planar double ring resonator (differential configuration) was proposed to exploit the effects of adsorbed molecular monolayers on the characteristics of TiO_2._ A nanotube membrane was positioned on one resonator (at the most sensitive region), while the other one monitored the sensor’s environment variations. They investigated three different monolayers and various levels of relative humidity (RH). In [54], a noncontact and non-intrusive flow sensor was proposed, based on a half-wavelength microwave resonator integrated with a PDMS-based membrane. The highest sensitivity recorded was 0.5 µL/min for the membrane (3 mm in diameter and 100 µm thick). 

## 6. Conclusions

This paper proposes a TE_20_-mode SIW resonator with an embedded layer of PDMS-based microfluidic channels as a dual-detection chemical sensor. The novelty of the proposed sensor is that it can simultaneously detect two chemicals using a single chip, compared with existing RF resonators, which use an array configuration for this purpose. The use of two asymmetric channels to non-uniformly perturb the symmetric E-field regions is the major contribution of this study. Additionally, sensing one analyte using two channels is more reliable than single-channel sensing. 

In the proposed TE_20_-mode SIW resonator, two symmetric yet distinct E-field regions collectively contribute to a single resonance frequency. To detect two chemicals, we loaded the two microfluidic channels, which were located on these high E-field regions. The loading of two of the same liquid, namely [ethanol, ethanol] and [DI water, DI water], yielded a distinct resonance frequency in each case, but failed to produce distinct resonance frequencies corresponding to the two extreme cases: [ethanol, DI water] and [DI water, ethanol]. As we already explained, the effective dielectric loading for the case of [E, DI] and [DI, E) remained the same. To achieve two distinct resonance frequencies corresponding to the two extreme cases, asymmetric microfluidic channels were envisaged, in which the symmetry of the E-field regions is non-uniformly disturbed. A meander-shaped channel with a large overlapped area gives rise to a high equivalent inductance and lowers the resonance frequency. A simple straight channel (21.36 µL) and a meander-shaped channel (48 µL) were utilized in this study. For future perspectives, we speculate that if one of the symmetric electric field regions caused by the TE_20_-mode SIW resonator is slightly displaced by geometry modifications, then two microfluidic channels with the same shape or dimensions may also perturb the effective permittivity of the dielectric substrate. We plan to investigate the simultaneous detection of multiple fluids with enhanced sensitivity in the future.

## Figures and Tables

**Table 1:** Design parameters of the TE_20_-mode SIW resonator (unit: mm).

Parameter	Value	Parameter	Value	Parameter	Value	Parameter	Value
a	60	d	1	j	4	m	5.25
b	40	e	22.2	k	0.5	n	0.8
c	30.8	g	10.85	l	1.5	o	1
p	1.8	i	6	u	8.45	q	50
r	0.75	s	11.15	v	0.8	w	0.9
h	0.6	h_film_	0.05	h_sub_	0.51		

**Table 2:** Comparison of the simulation and measurement results. The resonant frequencies and S11 measurements corresponding to empty channels and the four possible combinations of ethanol and DI water are provided.

Ch 1, Ch 2	Simu. f_r_ [GHz]	Simu. S11 [dB]	Meas. f_r_ [GHz]	Meas. S11 [dB]	Relative Error in f_r_ [%]
Air, Air	8.28	−25	8.26	−10.79	0.24
Ethanol, DI water	7.66	−16.87	7.669	−15.66	0.12
DI water, Ethanol	7.799	−11.48	7.795	−15.54	0.05
Ethanol, Ethanol	7.85	−8.67	7.84	−12.1	0.13
DI water, DI water	7.59	−14.76	7.60	−19	0.13

**Table 3:** Comparison of our proposed TE_20_-mode SIW resonator with recently proposed SIW chemical sensors.

Ref.	f_o_ [GHz]	Δf_max_ * [MHz]	Technology	Size ^†^	Sensing Application	Sensing
This work	8	660	SIW	2.37 λ_g_ × 1.58 λ_g_ (60 mm × 40 mm)	Ethanol	Dual
[7]	4.65	400	EMSIW	0.94 λ_g_ × 0.9 λ_g_ (35 mm × 30 mm)	Ethanol	Single
[28]	17.08	610	SIW	3 λ_g_ × 2.61 λ_g_ (35 mm × 30 mm)	Ethanol	Single
[34]	5	380	SIW	1.85 λ_g_ × 1.85 λ_g_ (75 mm × 75 mm)	Ethanol	Single
[47]	13.48	170	SIW	2.26 λ_g_ × 1.92 λ_g_ (33 mm × 28 mm)	Fibroblast cells	Single

* Δf_max_ represents the maximum frequency shift of the device, for instance, the shift corresponding to the cases in which air and an analyte or DI water are loaded. ^†^ λ_g_ represents the guided wavelength.

**Table 4:** Performance comparison of our proposed TE_20_-mode SIW resonator with other RF multichannel sensors.

Ref.	f_o_ [GHz]	Δf * [MHz]	ε_a_ **	ε_r_	S [MHz/ε_r_]	Physical Size	Electrical Size ^†^	Dielectric Constant of Substrate
This work	8	430	5	4	107.5	60 mm × 40 mm	2.37 λ_g_ × 1.58 λ_g_	2.2
[22]	3	170	6	5	34	35 mm × 32 mm	1.12 λ_g_ × 1.02 λ_g_	10.2
[23]	6.5	400	5	4	100	30 mm × 22 mm	1.13 λ_g_ × 0.86 λ_g_	3
[24]	0.87	110	14	13	8.46	86 mm × 62 mm	0.8 λ_g_ × 0.57 λ_g_	10.2

* Δf represents the shift in resonance frequency compared with the case of the empty resonator. Conventionally, frequency shifts in RF chemical sensors are compared with reference to DI water. However, because of insufficient data provided by some references, air was considered as the reference media. Therefore, Δf = f_air_ − f_a_, where f_air_ and f_a_ represent the resonance frequencies corresponding to air and the analyte, respectively, when present inside one or all channels. ** ε_a_ values are taken either from the reference mentioned or from [48], which is a standard document for the complex permittivity of dielectric reference liquids. ^†^ λ_g_ represents the guided wavelength.

**Table 5:** Qualitative analysis of our proposed TE_20_-mode SIW resonator with other RF chemical sensors, which have been proposed for dual/multichannel-detection.

Ref.	Substrate	Technology	Configuration	Noncontact	Independent Tuning	Detection
This work	RT/Duroid 5880	TE_20_ mode SIW	Unit cell	Yes	No	Dual
[22]	RT/Duroid 6010.2 LM	MM	Array	No	Yes	Multiple
[23]	RO3003	MM	Array	Yes	Yes	Dual
[24]	RO3010	MM	Array	Yes	No	Partially dual

Note: The configurations in [22,23,24] were four SRRs, three OSRRs, and two SRRs, respectively.
